# Effectiveness of Motivational Dental Care Interventions for Students in Vietnam

**DOI:** 10.3390/dj14060332

**Published:** 2026-06-01

**Authors:** Bau Minh Trinh, Toan Van Ngo, Tuan Manh Vu

**Affiliations:** School of Odonto-Stomatology, Hanoi Medical University, 1 Ton That Tung Street, Kim Lien, Hanoi 10000, Vietnam; trinhminhbau@hmu.edu.vn (B.M.T.); ngovantoan20281@gmail.com (T.V.N.)

**Keywords:** dental intrinsic motivation, extrinsic motivation, OHI-S, DMFT, dmft

## Abstract

**Background:** The prevalence of oral diseases in Vietnam has remained high in recent years, especially dental caries and gingivitis. Conventional dental education in schools is ineffective in maintaining good behavior. Evidence regarding motivational aspects of oral health education in primary school students is limited. Therefore, this study will evaluate the effectiveness of dental education interventions based on content that enhances motivation. **Methods:** A non-randomized two-cluster controlled before–after study was conducted in two primary schools in Hanoi, Vietnam, in 2024. Third-grade students were interviewed using a motivational questionnaire (DIM-S), then we examined the indicators OHI-S, DMFT (permanent teeth) and dmft (primary teeth) at the initial time (T1) and after 12 months (T2). Kim Lien School received a motivation-based education approach with content built upon the DIM-S guidelines; Khuong Thuong School received a traditional dental education approach. **Results:** At time point T1, extrinsic motivation predominated (86.41% in the control group; 95.95% in the intervention group). Mean motivation increased by 0.95 points in the intervention group (from 15.03 ± 1.56 to 15.98 ± 1.41; *p* < 0.001) compared with 0.17 points in the control group (*p* = 0.329); The difference is statistically significant (*p* < 0.001). The proportion of students with extrinsic motivation decreased by 8.91% in the intervention group (*p* < 0.001) but increased by 5.83% in the control group (*p* = 0.238); *p* = 0.003 when testing for differences between groups. The OHI-S index decreased in the intervention group (−0.31; *p* < 0.001) and increased in the control group (+0.14; *p* = 0.048); *p* < 0.001 when testing for differences in changes between the control and intervention groups. The DMFT index for permanent teeth increased in both groups, with no statistically significant difference (*p* = 0.178); the DMFT index for deciduous teeth decreased in both groups, with no statistically significant difference (*p* = 0.353). **Conclusions:** Motivation-based school dental education was associated with improved oral-care motivation and oral hygiene and with an increased percentage of students with intrinsic motivation after 12 months. However, maintaining student motivation still requires a combination of preventive measures to reduce oral disease incidence and longer follow-up.

## 1. Introduction

Dental caries and gingivitis are very common non-communicable diseases in children, causing pain, difficulty chewing, and affecting learning and quality of life [1]. The World Health Organization estimates that oral diseases still affect billions of people globally. In Vietnam, many studies show that the rate of tooth decay and poor oral hygiene among school-aged children remains high, indicating that the need for prevention and the challenge of changing students’ oral care behaviors are still significant [2,3].

School dental programs and dental education activities in Vietnamese schools are organized regularly, integrated into school health activities and typically focus on providing information (causes, consequences, and prevention) and instruction on proper brushing techniques [4]. However, interventions based primarily on “knowledge communication” often struggle to produce sustainable behavioral change. Children may understand but struggle to maintain daily routines, especially when lacking mechanisms to support autonomy and self-monitoring, or in other words, when they lack the motivation to drive the behavior. Recent systematic reviews suggest that school-based oral health education can improve knowledge, behavior, oral hygiene, and gingival health, but evidence for sustained long-term effects and caries prevention remains inconsistent [5,6,7]. These findings support the need for educational approaches that go beyond information delivery and address the motivational factors underlying daily oral-care routines.

In 1974, Becker developed the Health Belief Model, and motivation is a key factor in initiating and maintaining healthy behaviors [8,9]. In children, extrinsic motivation (reminders, supervision, rewards) can help initiate behavior, but intrinsic motivation (self-worth, autonomy, and responsibility) is often more closely related to long-term maintenance. Therefore, individualized interventions based on motivation are gaining attention as a new approach in dental education.

Motivation for oral care can be measured using the Dental Intrinsic Motivation Scale (DIM-S) proposed by Syrjälä et al. in 1992 [10]. In this study, we used 12 out of 15 items of DIM-S according to a scoring system used in some previous studies, with a total score ranging from 12 to 24; higher scores reflect higher intrinsic motivation. According to commonly used classification thresholds, a total score < 18 is classified as extrinsic motivation, and ≥18 is classified as intrinsic motivation [11].

In Vietnam, most research on school oral health focuses on prevalence and risk factors, while evidence on the role and level of motivation for oral care, as well as the effectiveness of dental education aimed at increasing intrinsic motivation, is limited.

Therefore, this study was conducted to: (1) describe the motivations for oral care and oral health status of 3rd grade students at two primary schools in Hanoi; (2) evaluate the effectiveness of motivation-based dental education (content design according to DIM-S criteria) compared to traditional dental education after 12 months, through changes in motivation score, intrinsic/extrinsic motivation ratio, and OHI-S, DMFT, dmft indices.

## 2. Materials and Methods

This study was approved by the Research Ethics Committee of Hanoi Medical University (Reference Number: IRB-VN01.001/IRB00003121/FWA 00004148). Before conducting the study, consent was obtained from the primary schools. Students agreed to participate, and parents signed consent forms.

### 2.1. Study Design and Participants

A non-randomized two-cluster controlled before–after study was conducted on third-grade students at two primary schools in Hanoi, Vietnam in 2024. Kim Lien Primary School was selected as the intervention group and Khuong Thuong Primary School as the control group. Data were collected at baseline (T1) and after 12 months (T2).

The study subjects were all third-grade students at two schools during the study year, with parental and student consent. Third-grade students were selected because they could read and understand questionnaires relatively well and were in the process of developing health care habits. Students who were absent at the time of data collection or who had systemic medical conditions that affected examinations/assessments (if any) were excluded.

Investigator calibration: Before data collection, investigators (dentists) received standardized diagnostic training based on the DMFT/dmft and OHI-S guidelines, assessed reliability among investigators, and repeated (test–retest) after 1–2 weeks, with a Kappa coefficient of 0.618.

Motivation Assessment: Students were interviewed directly using the DIM-S oral care motivation questionnaire [10]. The questions were translated and checked for semantic equivalence. Two bilingual English teachers independently translated the original English DIM-S items into Vietnamese. Their translations were compared for clarity, accessibility for children, and semantic equivalence with the original items. A reconciled Vietnamese version was then back-translated into English by two other translators who were not involved in the forward translation. The research team compared the back-translations with the original English version, discussed difficult terms and culturally sensitive wording, and finalized a Vietnamese version for administration. The Vietnamese DIM-S questionnaire administered in this study included 15 items. However, according to the scoring approach used in previous studies, only items 1–12 were used to calculate the total motivation score; each item had two options: “Agree/Disagree”. Scoring convention: Agree = 1 point; Disagree = 2 points; total score 12–24 [11]. A total score < 18 was classified as “extrinsic motivation” and ≥18 as “intrinsic motivation”. (Cronbach’s alpha = 0.668). Before implementation in the main school sample, the preliminary Vietnamese DIM-S was pilot-tested in 40 third-grade students who were not included in the main study sample. This test aimed to check the relationship between the Vietnamese wording and the original item meaning, assess whether 8–9-year-old students could understand each question and the Agree/Disagree response format, and identify possible interviewer-administration or data-recording errors. The questionnaire was administered using the same interviewer-administered procedure planned for the main study. The team assessed and adjusted the questionnaire. Data from the 40 pilot students were not included in the final analysis. This step was considered a linguistic/cognitive pre-test and error-verification procedure rather than a full independent psychometric validation study.

Because participants were 8–9-year-old children and some motivational constructs were abstract, procedural safeguards were applied to reduce cognitive burden. The questionnaire was interviewer-administered rather than self-completed; each item was read aloud using consistent wording, and the response format was limited to simple agree/disagree choices. Interviewers avoided leading explanations and asked students to answer independently.

Clinical examination: Following the interview, students underwent a dental examination at school under natural light/examination lamp conditions, using dental mirrors, probes, gauze, gloves, and sterile disposable or autoclaved instruments. The OHI-S index was recorded according to Greene & Vermillion [12]; the DMFT (permanent teeth) and dmft (deciduous teeth) indices were recorded according to the classic Klein–Palmer standard and the caries diagnostic guidelines applied in the study [13]. All data were recorded on standardized forms and double-entered to minimize errors.

Intervention: Before student sessions, teachers at both schools received oral health orientation from the research team covering common oral diseases, consequences, prevention, and correct oral hygiene practices. Both schools received the same 40 min in-class dental education session, incorporating presentations and guided brushing on models. The control group received traditional content (dental caries, gingivitis, nutrition, fluoride, and brushing technique). The intervention group received similar core topics, but the content was structured according to the DIM-S framework to increase autonomy and intrinsic motivation (details in Appendix B). The educational program was followed by a 40 min reinforcement session after six months at both schools.

Control of out-of-tracking: The study collaborated with homeroom teachers to create lists, assign identification numbers, schedule reassessment appointments, and organize make-up examinations for absent students (the reason for out-of-tracking was recorded as school transfer). Analysis was performed on students with sufficient T1 and T2 data; due to the very low out-of-tracking rate, the bias due to out-of-tracking was expected to be limited.

### 2.2. Sampling Technique and Data Collection

* Sample size in intervention studies:

Sample size was calculated using the Lwanga & Leme show formula for comparing two independent proportions for intervention studies [14]:

$$n=\frac{{\left\{z_{1-\alpha /2}\sqrt{2\bar{P}\left(1-\bar{P}\right)}+z_{1-\beta }\sqrt{P_{1}\left(1-P_{1}\right)+P_{2}(1-P_{2})}\right\}}^{2}}{{\left(P_{1}-P_{2}\right)}^{2}}$$

$z_{\left(1-\alpha /2\right)}$: confidence coefficient at the probability level 95% (=1.96)
$z_{1-\beta }$: statistical power (=80%)

P1 and P2 are the expected rates of extrinsic motivation in the control and intervention groups at T2.

P = (P1 + P2)/2

Assume the expected difference between P1 and P2 is 20%. According to calculations, the minimum sample size for each group is 97 students.

To account for loss of follow-up and due to the differing student sizes between the two schools, the study targeted sampling of all third-grade students who consented to participate: 105 students in Khuong Thuong (control group) and 249 students in Kim Lien (intervention group).

Motivation score calculation: using the total score of 12 questions (questions 1–12). According to the classification used in the reference study, a total score < 18 is categorized as extrinsic motivation-oriented, and ≥18 as intrinsic motivation-oriented.

## 3. Results

### 3.1. Characteristics of the Research Subjects

At time point T1, there were 354 third-grade students participating in the study (control group: 105; intervention group: 249).

After 12 months, the number of students who completed T1 and T2 follow-up was 103 in the control group and 247 in the intervention group (follow-up rates of 98.1% and 99.2%, respectively).

The majority of cases of students losing track of their progress are due to students transferring schools.

### 3.2. Comparison of the Changes After 12 Months

The changes in motivation scores and oral health indices from T1 to T2 are presented in Table 1.

### 3.3. Summary of Changes

At T1, the average motivation score in the control group was higher than in the intervention group (15.83 ± 1.54 vs. 15.03 ± 1.56), and the proportion of students with extrinsic motivation was very high in both groups (86.41% and 95.95%).

After 12 months, the intervention group increased their average motivation score by 0.95 points (*p* < 0.001) while the control group increased by 0.17 points (*p* = 0.329). The proportion of students with intrinsic motivation increased by 9.0% in the intervention group but decreased by 5.5% in the control group. The OHI-S index decreased in the intervention group (−0.31; *p* < 0.001) but increased in the control group (+0.14; *p* = 0.048). The DMFT index increased, and the dmft index decreased in both groups (both *p* < 0.001). However, the difference in the level of change in DMFT and dmft between the two groups was not statistically significant (*p* = 0.178 and *p* = 0.353).

The changes in motivation and dental caries prevalence are shown in Table 2.

Pairwise analysis (McNemar) showed that in the intervention group, the rate of extrinsic motivation decreased (*p* < 0.001) and the rate of intrinsic motivation increased (*p* = 0.000658), which were statistically significant; while in the control group, the change in the extrinsic/intrinsic ratio was not significant (*p* = 0.238 and *p* = 0.239). The rate of permanent caries increased in both groups (*p* < 0.01), while the rate of deciduous caries decreased in both groups (*p* < 0.05).

## 4. Discussion

### 4.1. Motivation for Oral Hygiene

The results showed that at baseline, third-grade students in both schools had primarily extrinsic motivation. This is a common characteristic in elementary school, where behaviors such as brushing teeth, limiting sugary foods, and visiting the dentist are largely maintained through reminders/supervision from parents and teachers. The initial rate of intrinsic motivation differed between the two schools (Khuong Thuong had a higher rate than Kim Lien), reflecting differences in school and family contexts that may exist due to the non-randomized design.

After 12 months, the intervention group showed increased motivation scores and decreased extrinsic motivation. This means that motivational intervention can help children transition from behaviors driven by external prompting to self-care behaviors; the results are consistent with motivational theory and some previous studies.

For the *p*-value, the “comparison Δ” test examined the difference in the level of change between the two groups (difference-in-differences approach). The results, *p* < 0.001 for motivation score and *p* = 0.003 for extrinsic/intrinsic motivation ratio, showed that the improvement in the intervention group was greater than in the control group, indicating the impact of motivation-based education.

Our results are consistent with several studies conducted in various schools, where dental education interventions resulted in improved oral hygiene and changes in oral care habits. A randomized trial by Wu L et al., using motivational interviewing, showed greater effectiveness than conventional dental education in reducing new caries [15]. A study by Aleksejūniene J on self-determination theory focusing on intrinsic motivation also showed improved self-care in adolescents, although the sustained effect after 12 months was limited [16].

### 4.2. Current Situation of Oral Diseases

At baseline, oral health and hygiene indicators showed a high burden of disease among third-grade students. Notably, the average OHI-S of the intervention group was higher than that of the control group (poorer oral hygiene), suggesting initial differences between the two groups.

Regarding the relationship between motivation and caries status, DMFT/dmft is a “cumulative” index over time, while motivation is measured at the time of the survey and can fluctuate depending on the context. Therefore, the cross-sectional association between motivation score and DMFT/dmft may not be high, especially when the intrinsic motivation group accounted for a small proportion in T1.

### 4.3. Intervention Effectiveness After 12 Months

Motivation-based interventions appeared to show a change, with an 8.91 percentage point decrease in extrinsic motivation and a 9.0 percentage point increase in intrinsic motivation in the intervention group (both significant according to McNemar), while the control group showed the opposite trend. This is consistent with the hypothesis that integrating content according to DIM-S objectives helps children understand “why” oral hygiene is necessary and increases their sense of personal responsibility.

Regarding oral hygiene, the intervention group experienced a mean decrease in OHI-S of 0.31 points (*p* < 0.001), while the control group saw an increase of 0.14 points (*p* = 0.048). In practical terms, these results suggest that structured, motivational education, incorporating instruction on brushing techniques, can improve brushing behavior and plaque removal in school settings.

However, with regard to caries indices, DMFT increased in both groups, and the difference in the increase was not statistically significant (*p* = 0.178). This may reflect (1) the eruption of new permanent teeth at age 8–9, which increases the risk of caries in the eruption phase; (2) caries is a multifactorial disease, and education alone is unlikely to produce sufficiently large changes in 12 months without accompanying preventive clinical interventions (fluoride varnish, fissure sealants) and nutritional interventions. This result is consistent with previous reviews on educational interventions that can improve oral hygiene, but the impact on caries prevention has been inconsistent [17].

The dmft index decreased in both groups, and the difference in the degree of decrease was not statistically significant (*p* = 0.353). Part of the decrease in dmft may be due to the replacement of deciduous teeth with permanent teeth during the follow-up period, so it cannot be simply interpreted as a reduction in disease due to intervention. No statistically significant between-group differences were observed for changes in DMFT or dmft over 12 months. Improvements in motivation and OHI-S are primarily a result of behavior and oral hygiene, while caries indices are cumulative clinical outcomes. DMFT and dmft may not be sensitive enough to detect the impact of education alone over a relatively short follow-up period, especially in the mixed dentition stage. Therefore, to assess the impact on caries, the “incidence” index, or the number of new caries over time, combined with a longer follow-up period, would be more accurate than DMFT/dmft.

In summary, the observed benefits were mainly related to the motivation score and oral hygiene. In contrast, no statistically significant between-group differences were observed for DMFT or dmft changes. A more rational approach is to integrate motivational education with preventive measures.

## 5. Limitations of the Study

The study has several limitations. First, the non-randomized design, consisting of only two schools (two clusters), may introduce sampling bias and make it difficult to adequately control for confounding factors within each cluster; therefore, generalizability is limited. Second, the two groups have background differences (e.g., OHI-S), which may affect impact estimates even when compared by magnitude of change. Third, motivation was measured using a self-reported questionnaire in children, so it may be influenced by social bias and the level of comprehension of the questions. Fourth, during the 12 months of follow-up, children’s tooth replacement and eruption of new permanent teeth cause physiological fluctuations in DMFT/dmft, making the cumulative index less sensitive to detect the impact of education. Further studies should consider a randomized cluster design, longer follow-up, and the use of a new caries incidence index along with clinical preventive interventions.

## 6. Conclusions

This non-randomized two-cluster controlled before–after study among third-grade students from two primary schools in Hanoi showed that initial motivation for oral care was primarily extrinsic, and the average motivation score was low. After 12 months, motivation-based dental education structured according to DIM-S was associated with higher motivation scores and a higher percentage of students classified as intrinsically motivated than traditional dental education. Simultaneously, oral hygiene (OHI-S) improved in the intervention group, while it tended to worsen in the control group. However, the observed benefits of the intervention were mainly behavioral and hygiene-related; changes in caries indices (DMFT, dmft) were not significantly different between the two groups, suggesting that education alone was insufficient to reduce caries within 12 months. Therefore, school dental programs should combine intrinsic motivation-oriented education with clinical prevention measures and nutritional interventions for comprehensive effectiveness.

## Figures and Tables

**Table 1:** Mean motivation scores and oral indices at time points T1 and T2 (after 12 months).

Variable	Group	T1 (Mean ± SD)	T2 (Mean ± SD)	Δ (T2-T1)	*p* (In Group)	*p* Compares Δ (Intervention vs. Control)
Motivation average score	Control	15.83 ± 1.54	15.99 ± 1.19	0.17	0.329	<0.001
Motivation average score	Intervention	15.03 ± 1.56	15.98 ± 1.41	0.95	<0.001 *	
Motivation average score	2 groups	15.26 ± 1.59	15.98 ± 1.35	0.72	<0.001 *	
OHI-S	Control	1.12 ± 0.57	1.26 ± 0.55	0.14	0.048	<0.001
OHI-S	Intervention	1.70 ± 0.45	1.39 ± 1.14	−0.31	<0.001 *	
OHI-S	2 groups	1.53 ± 0.55	1.35 ± 1.01	−0.18	<0.001 *	
DMFT	Control	1.14 ± 1.39	2.04 ± 1.66	0.90	<0.001	0.178
DMFT	Intervention	0.66 ± 1.20	1.44 ± 1.94	0.78	<0.001	
DMFT	2 groups	0.81 ± 1.29	1.63 ± 1.88	0.81	<0.001	
dmft	Control	3.64 ± 2.96	2.81 ± 2.50	−0.83	<0.001	0.353
dmft	Intervention	3.85 ± 2.98	3.28 ± 2.92	−0.57	<0.001	
dmft	2 groups	3.79 ± 2.97	3.14 ± 2.81	−0.65	<0.001	

Note: * indicates statistically significant results (p < 0.05).

**Table 2:** Changes in the proportion of students with extrinsic/intrinsic motivation and the rate of dental caries at T1 and T2.

Variable	Group	T1 (%)	T2 (%)	Δ (%)	*p* (McNemar)	*p* for Comparison of Change Between Groups
Extrinsic motivation	Control	86.41	92.23	5.83	0.238	0.003
Extrinsic motivation	Intervention	95.95	87.04	−8.91	<0.001 *	
Extrinsic motivation	2 groups	93.14	88.57	−4.57	0.045	
Permanent tooth decay	Control	44.7	65.0	20.4	<0.001 *	0.126
Permanent tooth decay	Intervention	25.1	37.2	12.1	0.001 *	
Permanent tooth decay	2 groups	30.9	45.4	14.6	<0.001 *	
Primary tooth decay	Control	77.7	67.0	−10.7	0.027	0.667
Primary tooth decay	Intervention	81.4	68.0	−13.4	<0.001	
Primary tooth decay	2 groups	80.3	67.7	−12.6	<0.001	

Note: * indicates statistically significant results (p < 0.05).

## Data Availability

The data presented in this study are available on request from the corresponding author due to privacy and ethical restrictions.

## Appendix A. Motivation Survey Questionnaire and Scoring Method

**Table A1:** Motivation survey questionnaire [10].

	Question	Agree	Disagree
1	I think it’s not important to retain one’s own teeth, because extracted teeth can be replaced by prostheses.		
2	I think the most important thing in dental care is to avoid pain.		
3	I think it’s more important to fill front teeth than back teeth.		
4	I think I would prefer to have painful teeth extracted rather than retaining them.		
5	I have reduced my eating of sweet snacks (e.g., bonbons, lozenges, ice scream, cakes) in order to stop my teeth decaying.		
6	When I notice a broken filling, I tell my parents to go to the dentist.		
7	I brush my teeth more carefully than usual when I am going to the dentist.		
8	I can’t go to bed if I haven’t brushed my teeth.		
9	I often brush the outside surfaces of my teeth better than the inside surfaces.		
10	It gives me pleasure to brush my teeth carefully.		
11	I watch my teeth spontaneously and contact the dentist when necessary.		
12	I can ask my dentist about dental care as much as I need.		
13	I do not get enough detailed information about dental care.		
14	The dentist is mostly responsible for my dental health.		
15	The dentist recalls me once a year.		

Scoring method:

Calculate the total score for questions 1 to 12.

- Agree = 1 point.
- Disagree = 2 points

Total score < 18 points: student has extrinsic motivation, ≥18 = intrinsic motivation

## Appendix B. Intervention Method: Both Groups Received In-Class Dental Education for Students and Teachers, Lasting One 40-Min Lesson with a Second Session After 6 Months

1. Control group
  1.1. Oral health education
    - Dental caries: causes, consequences, and prevention
    - Gingivitis: causes, consequences, and prevention
    - Nutrition
    - Effects of Fluoride
  1.2. Instructions for brushing teeth on a model.
2. Intervention Group: Oral health education
  - Keep the same themes as the control group.
  - The content is designed based on the DIM-S questionnaire.

**Table A2:** Motivation-based intervention content mapped to DIM-S questionnaire items.

Question	Corresponding Content
I think it’s not important to retain one’s own teeth, because extracted teeth can be replaced by prostheses.	The role of teeth: chewing, esthetics.
I think the most important thing in dental care is to avoid pain.	Benefits of oral care: helps keep teeth pain-free and healthy.
I think it’s more important to fill front teeth than back teeth.	The structure and surfaces of molars and incisors. Cleaning must cover all surfaces of the teeth; all teeth are important.
I think I would prefer to have painful teeth extracted rather than retaining them.	The role of oral disease prevention.
I have reduced my eating of sweet snacks (e.g., bonbons, lozenges, ice cream, cakes) in order to stop my teeth decaying.	A diet that reduces the frequency of consuming sweets, and a game designed to sort images of foods harmful to teeth.
When I notice a broken filling, I tell my parents to go to the dentist.	The benefits of dental fillings.
I brush my teeth more carefully than usual when I am going to the dentist.	The benefits of brushing teeth for oral health.
I can’t go to bed if I haven’t brushed my teeth.	When to brush teeth.
I often brush the outside surfaces of my teeth better than the inside surfaces.	In the brushing technique demonstration, students practice on a model, brushing each area about 10 times and counting in front of the class.
It gives me pleasure to brush my teeth carefully.	Students demonstrate brushing their teeth in class and talk about how it feels.
I watch my teeth spontaneously and contact the dentist when necessary.	The benefits of regular check-ups include early detection of oral diseases.
I can ask my dentist about dental care as much as I need.	Show a fun Q&A session for students.
I do not get enough detailed information about dental care.	Summarize the content for students and encourage them to repeat it.
The dentist is mostly responsible for my dental health.	The dentist’s role is supportive.
The dentist recalls me once a year.	Regular check-ups every 6–12 months.
